# Thymosin *β*4 Protects against Cardiac Damage and Subsequent Cardiac Fibrosis in Mice with Myocardial Infarction

**DOI:** 10.1155/2022/1308651

**Published:** 2022-06-03

**Authors:** Fei Wang, Yajuan He, Naijuan Yao, Litao Ruan, Zhen Tian

**Affiliations:** ^1^Department of Ultrasound, The First Affiliated Hospital of Xi'an Jiaotong University, Xi'an, Shaanxi Province, China; ^2^Department of Infectious Diseases, The First Affiliated Hospital of Xi'an Jiaotong University, Xi'an, Shaanxi Province, China

## Abstract

**Background:**

Inflammation is a critical factor in the development and progression of myocardial infarction and cardiac fibrosis. Thymosin *β*4 (T*β*4) alleviates the disease process via protective antioxidant and anti-inflammatory mechanisms. Although T*β*4 has been shown to have a protective effect in myocardial infarction, its impact on cardiac fibrosis has not been well reported. In this study, we evaluated the influence of exogenous T*β*4 on myocardial infarction and cardiac fibrosis and explored the possible underlying mechanism.

**Methods:**

Real-time quantitative reverse-transcription PCR (qRT-PCR), immunohistochemistry (IHC), and Western blot were used to analyze T*β*4 expression in acute myocardial infarction (AMI) cardiac tissues. The effects of intraperitoneal adeno-associated virus-T*β*4 (AAV-T*β*4) on ligation-induced AMI in mice were studied using cardiac function parameters, and RT-PCR, Western blot, HE staining, Masson staining, and IHC were used to assess the degree of myocardial fibrosis. The effects of T*β*4 were confirmed in vitro using mouse cardiac myocytes and myofibroblasts.

**Results:**

T*β*4 was shown to be significantly elevated in mice AMI cardiac tissues. In mice, AAV-T*β*4 induced exogenous expression of T*β*4 significantly reduced oxidative damage, inflammation, cardiac dysfunction, and fibrosis. H_2_O_2_ inhibited mitophagy and increased inflammation in mouse cardiac myocytes via oxidative stress, and T*β*4 substantially reduced mitophagy inhibition and inflammasome activation in myocytes caused by H_2_O_2_. Furthermore, T*β*4 decreased cardiac myofibroblast growth and reduced TGF-*β*1-induced activation.

**Conclusions:**

AAV-T*β*4 induced expression of T*β*4 reduced inflammation, heart damage, and eventual fibrosis in vivo. T*β*4 helped to reduce oxidative stress, promote mitophagy, and alleviate inflammation and fibrosis. Exogenous supplementation of T*β*4 might be a promising therapeutic agent for treating myocardial infarction as well as cardiac fibrosis.

## 1. Introduction

Myocardial fibrosis is characterized by a significant accumulation of extracellular matrix (ECM) in the myocardium [1]. Alterations of the cardiac ECM and cardiac remodeling play major roles in the development of cardiac fibrosis [2]. At the site of acute myocardial infarction (AMI), the sudden loss of a large number of cardiomyocytes triggers an inflammatory reaction, ultimately leading to the replacement of necrotic myocardium with a collagen-based network [3, 4].

The etiology of cardiac fibrosis has been linked to uncontrolled oxidative stress and the consequent release of proinflammatory and profibrotic cytokines [5]. This causes cardiac myocyte injury, which is followed by cardiac myofibroblast proliferation, ECM protein deposition, and interstitial remodeling [6]. Therapy for myocardial infarction and cardiac fibrosis frequently employs antioxidant, anti-inflammatory, and antifibrotic medicines. However, no therapeutic strategy has been developed that ensures damaged tissue reversal [7–9].

Thymosin 4 (T*β*4) is a 43-amino acid protein that belongs to the *β*-thymosin family, which is highly conserved [10]. It is released into body fluids such as tears, saliva, and plasma to regulate cell functions [11, 12]. T*β*4 has been associated with wound healing, inflammation, fibrosis, and tissue regeneration, with recent studies suggesting that T*β*4 can help prevent inflammation and fibrosis in the eye, skin, lung, and liver [12–15]. T*β*4 is a potent protective factor that can protect against myocyte damage, promote myocyte regeneration, and inhibit heart inflammation [16, 17]. Heart injury and inflammation are the precursors of myocardial infarction and cardiac fibrosis. Therefore, we propose that T*β*4 might have an antifibrotic function in the heart.

Autophagy is a highly conserved mechanism that maintains homeostasis by catabolizing cytoplasmic components, such as defective proteins and organelles [18]. Autophagy contributes to the end of NLRP3 inflammasome activation by targeting reactive oxygen species ROS-producing mitochondria. The process by which mitochondria are degraded by autophagy is called mitophagy [19, 20]. T*β*4 has been found in recent research to reduce inflammation by promoting autophagosome formation and membrane remodeling during autophagy [21], and T*β*4 may potentially protect against oxidative stress by increasing the activity of the antioxidant enzyme Cu-Zn superoxide dismutase (SOD) [22]. However, no research has investigated whether mitophagy controls inflammation through T*β*4 during myocardial infarction and cardiac fibrosis.

In this study, we first determined the expression of T*β*4 in infarcted mouse heart tissues. Next, the effects of adeno-associated virus- (AAV-) mediated ectopic T*β*4 expression on ligation-induced AMI were observed. Moreover, the underlying mechanisms for the antifibrotic effects of T*β*4 were explored by in vivo and in vitro experiments.

## 2. Materials and Methods

### 2.1. Preparation of Recombinant AAV

An AAV Helper-Free System (Cell Biolabs, San Diego, CA, USA) was used to create self-complementary recombinant adeno-associated virus.

The pscAAV-T*β*4 plasmid was created by inserting the coding DNA of human T*β*4 (GenBank NM_021109.3) into pscAAV-MCS. pscAAV-T*β*4, pHelper, and pAAVRC5 were cotransfected into AAV-293 cells using polyethyleneimine (PEI) to produce recombinant AAV carrying T*β*4 (AAV-T*β*4). As a control virus, recombinant AAV containing LacZ (AAV-LacZ) was created. The cells were then collected 72 hours after transfection for viral particle separation, purification, and quantitative examination.

The titers and abundance of recombinant AAV (rAAV) in the heart were determined using TaqMan Universal PCR Master Mix (Applied Biosystems, Foster City, CA, USA). The following primers were used to target the CMV promoter region: 5′-CGTATTAGTCATCGCTATTACCATGGT-3′ (forward) and 5′-AGACTTGGAAATCCCCGTGAGT-3′ (reverse). 5′-6FAM-AACCGCTATCCACGCCCATTGATG-TAMRA-3′ was the probe's sequence. The standard curve approach was used to examine the acquired data.

### 2.2. Animals

Specific pathogen-free, 6-week-old male C57BL/6I mice, weighing 25–30 g was obtained from the Experimental Animal Center, School of Medicine, Xi'an Jiaotong University. The mice were kept in pathogen free environments with a 12/12 h light/dark cycle, consistent temperature (23 ± 2°C), and humidity, as well as free access to water and normal laboratory food. Before beginning the tests, all of the mice were acclimatized to the abovementioned circumstances for one week. All attempts were made to keep the mice as comfortable as possible.

**Figure 1:**
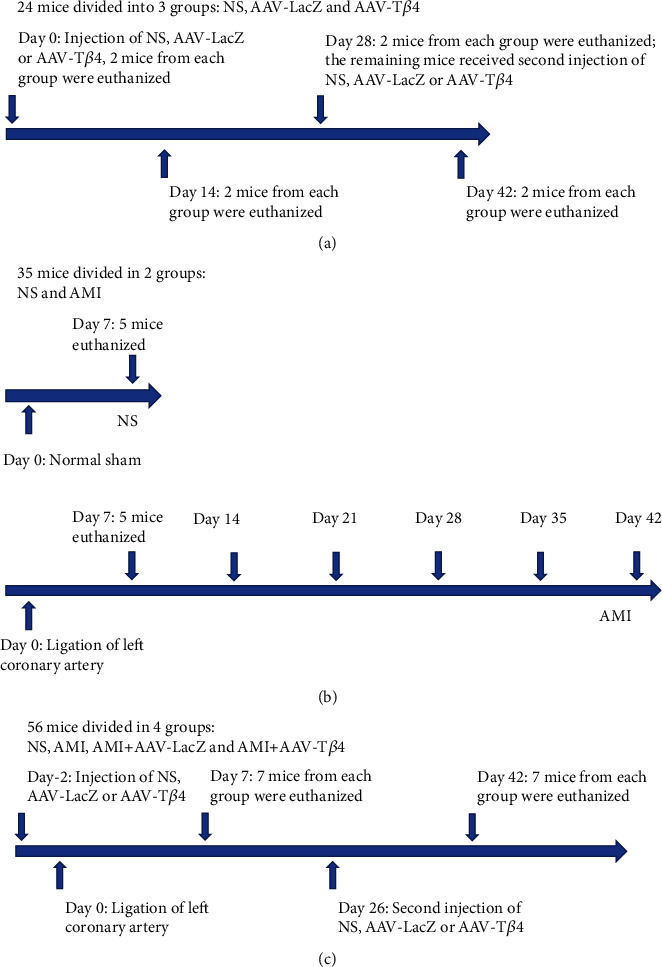
Timeline for the experimental procedures. Procedures for repeated intraperitoneal AAV injections in mice (a). Procedures for ligation-induced AMI mice models (b). Procedures for AAV-T*β*4 in AMI mice.

To investigate the transduction effectiveness of repeated intraperitoneal (IP) rAAV injections, 24 mice were separated into three groups: PBS, AAV-LacZ, and AAV-T*β*4. On day 0, mice in the PBS group received PBS, mice in the AAV-LacZ group received AAV-LacZ (4 × 10^10^ viral genome (vg)), and mice in the AAV-T*β*4 group received AAV-T*β*4 (4 × 10^10^ vg). On days 14 and 28, two mice from each group were selected at random and euthanized. On day 28, the remaining mice were injected again with AAV-LacZ or AAV-T*β*4, and on day 42, they were euthanized. The hearts of the euthanized mice were excised for further examination (Figure 1(a)).

### 2.3. AAV-Mediated T*β*4 Expression upon Ligation-Induced AMI

Permanent closure of the left coronary artery of mice was performed as previously reported to generate the ligation-induced AMI mouse model [23]. To examine the expression of T*β*4 in mouse hearts following ligation, 35 mice were divided into normal sham (NS, *n* = 5) and AMI (*n* = 30) groups. Five AMI mice were euthanized on days 7, 14, 21, 28, 35, and 42, whereas all NS mice were euthanized on day 7 (Figure 1(b)). Hematoxylin and eosin (HE) and Masson staining, Western blot, and other assays were performed on mouse hearts.

To evaluate the effects of T*β*4 on myocardial infarction and fibrosis, 56 mice were divided equally into four groups: NS, AMI, AMI + AAV-LacZ, and AMI + AAV-T*β*4. The AAV groups received a single IP injection of AAV (AAV-LacZ or AAV-T*β*4, 4 × 10^10^ vg) for the first time, whereas the other two groups received an equal quantity of normal saline. The animals received sham or permanent closure of the left coronary artery two days later (day 0). Seven mice from each group were euthanized on the seventh day. The remaining mice received a second IP injection of AAV or normal saline on day 26 (four weeks after the first viral delivery) and were euthanized on day 42, with the hearts excised and serum collected for further investigation (Figure 1(c)).

### 2.4. Measurement of Malondialdehyde (MDA) and Myeloperoxidase (MPO)

Commercially available kits (Nanjing Jiancheng Bioengineering Institute, Nanjing, China) were used to detect MDA content and MPO activity in mouse cardiac tissue in line with the manufacturer's instructions.

### 2.5. Measurement of Hydroxyproline Content

Commercially available kits (Nanjing Jiancheng Bioengineering Institute, Nanjing, China) were used to determine cardiac hydroxyproline content, which was done according to the manufacturer's instructions.

### 2.6. Mouse Cardiac Myocyte and Myofibroblast Isolation

#### 2.6.1. Cardiac Myocyte Isolation

Kasten's approach was modified to isolate mouse ventricular myocytes, as previously described [24]. The hearts were extracted from 8-week-old mice that had been sedated with ether and then decapitated. Ventricular tissues were digested overnight in Hanks' balanced salt solution (HBSS) (Ca^2+^ free; GIBCO BRL, Gaithersburg, MD) with 0.1 percent trypsin (Worthington Biochemical Corp., Freehold, NJ) at 4°C. Repeated digestions of the tissue in 10 mL of 0.1% collagenase in HBSS were used to recover the ventricular cells. Following each digestion, the supernatants were centrifuged for 3 min at 100 g (4°C). The pellets were resuspended in ice-cold HBSS, pooled, and centrifuged for 4 min at 100 g (4°C). To enrich myocytes and reduce contamination by nonmuscle cells, cells were resuspended in Dulbecco's modified Eagle medium (DMEM) containing 7% heat-inactivated fetal bovine serum (FBS) and preplated twice in T80 flasks (Nunc, Inc., Naperville, IL) for 75 min.

#### 2.6.2. Cardiac Myofibroblast Isolation

Collagenase digestion was used to isolate cardiac fibroblasts, as previously described [25]. In brief, 8-week-old mouse ventricles were chopped into pieces and digested with 0.1% collagenase I for 10 min at 37°C with steady stirring. The supernatants were collected, and the digestion cycle was repeated 6–10 times until all of the tissue was completely dissolved. The cells were pelleted and grown in DMEM supplemented with penicillin and streptomycin and 10% inactivated FBS. The adherent fibroblasts were grown to confluence after the unattached cells were removed 2 h later.

### 2.7. Measurement of JC-1 Staining and CellROX Evaluation

The JC-1 staining (Thermo Fisher Scientific, Waltham, MA, USA) method was utilized, which reveals red fluorescence in normal mitochondrial potential and green fluorescence in damaged mitochondrial potential. In 6-well plates, mouse cardiac myocytes were seeded and treated with 400 *μ*M H_2_O_2_ for up to one hour. JC-1 was added to each well at a concentration of 10 mg/mL and incubated in the dark for 10 minutes at 37°C. The cells were collected and analyzed using a flow cytometer.

The CellROX deep red oxidative stress reagent (Thermo Fisher Scientific, Waltham, MA, USA) was utilized, which is nonfluorescent in a reduced state but produces a strong fluorogenic signal when oxidized. In 6-well plates, mouse cardiac myocytes were seeded and treated with 100 *μ*M H_2_O_2_ for up to 2 hours. Each well received 10 *μ*g/mL CellROX deep red and was incubated for 15 minutes. The cells were collected and analyzed using a flow cytometer.

### 2.8. Measurement of IL-1*β*, IL-6, and TNF-*α*

The levels of IL-1*β*, IL-6, and TNF-*α* were determined by enzyme-linked immunosorbent assay (ELISA) using commercially available kits (eBioscience, San Diego, CA, USA) and following the manufacturer's instructions.

### 2.9. Cell Culture, Proliferation Assay, and Reagent Treatment

The cardiac myocytes and myofibroblasts of mice were grown at 37°C in a 95% air, 5% CO_2_-humidified environment. The cells were trypsinized, and 5 × 10^5^ cells/well were seeded onto 6-well plastic plates with H_2_O_2_ (0, 100, 200, and 400 *μ*M), NAC (10 mM), FCCP (10 mM), oligomycin (10 mM), or TGF-1 (5 ng/mL).

The cells were seeded at 500 cells/well onto 96-well plates and allowed to adhere for 24 h. After that, the cells were treated with T*β*4 at various doses (0, 75, or 150 nM) and incubated for another 72 h. The viability of the cells was determined using the cell counting kit-8 (CCK-8) (Dojindo, Kyushu, Japan) test at 24, 48, and 72 h, according to the manufacturer's instructions. There were three groups in the experiment: blank group, 75 nM T*β*4, and 150 nM T*β*4. Cells from different treatment groups were adjusted to a concentration of 1 × 10^5^ cells/mL and seeded in a 96-well plate at 100 *μ*L/well. Cells were seeded in triplicate for each treatment group. The cells were incubated at 37°C in a 95% air, 5% CO_2_-humidified environment for the appropriate duration. 10 *μ*L CCK-8 solution was added to each well, and the cells were incubated for 1–4 h. The absorbance at 450 nm was measured using a plate reader.

### 2.10. Western Blot

RIPA Lysis Buffer supplemented with complete EDTA-free protease inhibitor cocktail tablets (Roche Applied Science, Basel, Switzerland) and phosphatase inhibitor cocktail tablets (Sigma-Aldrich) were used to extract proteins from cells and mice kidney tissues. SDS-PAGE gels were used to load protein samples, which were then transferred to PVDF membranes. After blocking for 2 h at room temperature in 5% evaporated milk in TBS+ 0.1% Tween 20, the membranes were incubated overnight at 4°C with the appropriate primary antibodies in 5% evaporated milk in TBS+ 0.1% Tween 20. The principal antibodies utilized were as follows: anti-Thymosin *β*4 (ab167650, Abcam, Cambridge, UK), anti-*α*-smooth muscle actin (SMA) (#56856, Cell Signaling Technology, Danvers, MA, USA), IL-1*β* (#12703, Cell Signaling), PINK1 (#6946, Cell Signaling), anti-Tom40 (H-300, Santa Cruz Biotechnology, Santa Cruz, CA), and *β*-actin as a loading control (#4970, Cell Signaling). A chemiluminescent substrate was used to create the signals, which were then viewed using X-ray films.

### 2.11. Immunohistochemistry

On chosen heart sections, immunoreactions were conducted. Antigens were identified by the appropriate primary antibodies: anti-Thymosin *β*4 (ab167650, Abcam, Cambridge, UK) and anti-*α*-SMA (#56856, Cell Signaling Technology, Danvers, MA, USA), which were then detected by secondary antibodies. After that, the slides were examined using a Nikon Eclipse microscope (Tokyo, Japan) and coupled to a digital camera.

### 2.12. Statistical Analysis

The data is presented as a mean ± standard deviation. The SPSS software 13.0 (SPSS, Inc., Chicago, IL, USA) was used for statistical analysis. The normality and homogeneity of the variance were assessed using the Shapiro-Wilk test and the Levene statistic, respectively. Mann–Whitney *U* tests or *t*-tests were employed to assess differences between two groups on this basis. Pearson or Spearman correlation tests were used to examine correlations between two quantitative groups. For comparisons between two groups, the *χ*2 test was utilized. *P* values < 0.05 were considered statistically significant.

## 3. Results

### 3.1. The Expression of T*β*4 Is Increased in Mouse Cardiac Tissues

**Figure 2:**
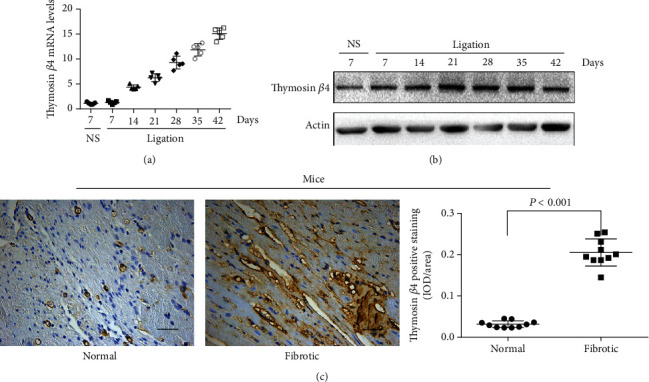
Expression of T*β*4 in infracted mouse heart tissues. The expression of T*β*4 in mouse heart tissues at both mRNA (a) and protein (b) levels. Immunohistochemistry of T*β*4 in normal and infracted mouse heart tissue (c). Scare bars = 100 *μ*m.

In AMI mice, RT-PCR and Western blot consistently demonstrated that T*β*4 was markedly elevated starting from day 7 after ligation and increased thereafter (Figures 2(a) and 2(b)). The increased expression of T*β*4 was also confirmed by IHC (Figure 2(c)), resulting in a significant increase in the average integrated optical density compared to that of normal tissues.

### 3.2. Intraperitoneal Injection of Adeno-Associated Virus Transduces Cardiac Heart Tissue

**Figure 3:**
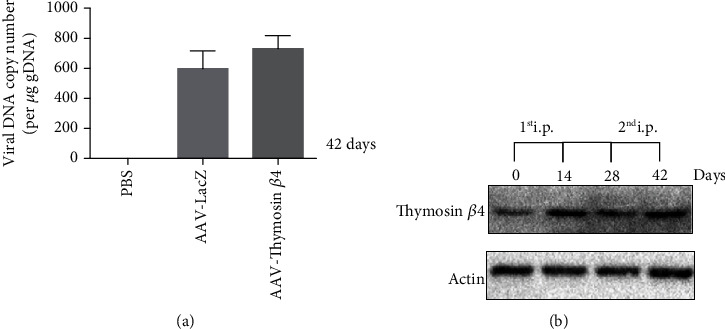
Expression of T*β*4 in mouse heart tissues after intraperitoneal (i.p.) administration of an adeno-associated virus containing Thymosin 4. qRT-PCR revealed the expression of vector DNA in NC, AAV-LacZ, and AAV-T*β*4 mice (a). Western blot revealed the expression of T*β*4 in the mouse heart tissues (b).

To assess the transduction effectiveness of recombinant adeno-associated virus, we used qRT-PCR to determine the quantity of vector DNA in mouse cardiac tissue. qRT-PCR revealed the expression of vector DNA in AAV-LacZ and AAV-T*β*4 mice compared to NS mice, as shown in Figure 3(a). Western blot revealed higher expression of T*β*4 on days 14 and 42, 14 d after the injection of recombinant adeno-associated virus days 0 and 28. Moreover, the expression of T*β*4 on day 42 was comparable to the expression observed on day 14, indicating that repeated injection of the recombinant adeno-associated virus could be used to achieve prolonged ectopic expression (Figure 3(b)).

### 3.3. AAV-T*β*4 Protects Mice from Ligation-Induced Infarction, Oxidative Stress, and the Inflammatory Responses

**Figure 4:**
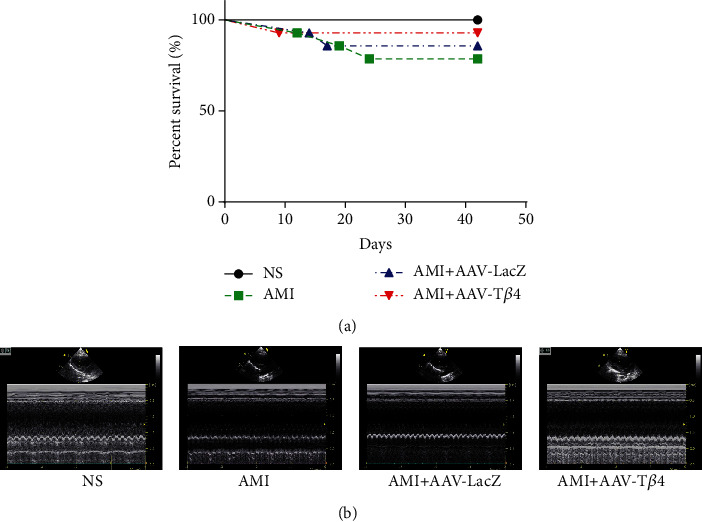
AAV-T*β*4 alleviates ligation-induced heart infarction in mice survival curve of mice in the NS, AMI, AMI + AAV-LacZ, and AMI + AAV-T*β*4 groups (a). Representative M-mode echocardiogram was conducted on conscious animals in cardiac function on day 42 after ligation (b).

Exogenous T*β*4 reduced the ligation-induced death rate; as all mice survived in the NS group, three mice died in the AMI group, two mice died in the AMI + AAV-LacZ group, and one mouse died in the AMI + AAV-T*β*4 group by day 42 (Figure 4(a)).

Using echocardiography, a clinically relevant measurement, we demonstrated that AAV-T*β*4 improved the cardiac function of AMI mice. Compared with the AMI or AMI + AAV-LacZ groups, exogenous T*β*4 reduced AMI associated changes in echocardiographic measurements, including the left ventricular internal dimension (LVID) and left ventricular ejection fraction (LVEF) at day 42 (Figure 4(b) and Table 1).

On day 7 and day 42, we discovered that the AMI + AAV-T*β*4 group had lower tissue MDA content than the AMI or AMI + AAV-LacZ groups (Table 2). MPO activity in the tissues, a marker of oxidative stress and neutrophil infiltration, was higher in the AMI group but was reduced by AAV-T*β*4 (Table 2).

TNF-*α*, IL-1*β*, and IL-6 in serum were evaluated to further investigate the anti-inflammatory function of T*β*4 in AMI mice; the results revealed that AAV-T*β*4 considerably reduced the expression of these inflammatory mediators (Table 3).

Exogenous T*β*4 alleviated oxidative stress and the inflammatory response in vivo during the pathogenesis of AMI.

### 3.4. AAV-T*β*4 Attenuated Myocardial Fibrosis in Mice

**Figure 5:**
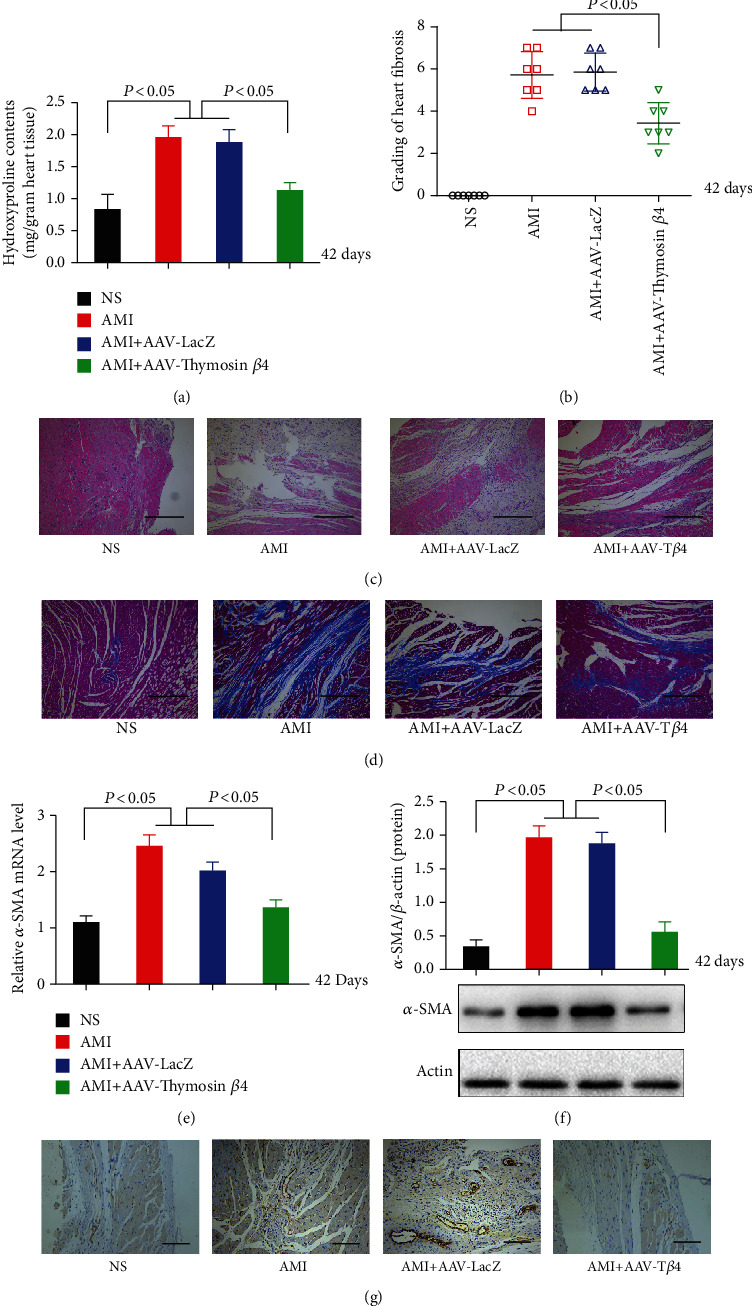
AAV-T*β*4 alleviates ligation-induced heart fibrosis in mice. Detection of cardiac hydroxyproline content (a), fibrosis score (b), HE staining (c), and Masson staining (d) in the NS, AMI, AMI + AAV-LacZ, and AMI + AAV-T*β*4 groups. Expression of *α*-SMA in the mouse heart tissues detected by rt-PCR (e), Western blot (f), and immunohistochemistry (g). Scare bars = 100 *μ*m.

Exogenous T*β*4 reduced ligation-induced heart fibrogenesis in mice, as evidenced by a decreased cardiac hydroxyproline content (Figure 5(a)), milder heart structural damage (Figure 5(c)), reduced Masson-positive staining (Figure 5(d)), and a lower fibrosis score (Figure 5(b)) in the AAV-T*β*4 group compared with the AMI and AMI + AAV-LacZ groups, 42 d following ligation. In comparison to that of the AMI and AMI + AAV-LacZ groups, AAV-T*β*4 dramatically reduced ligation-induced excess expression of *α*-SMA in the mouse heart, as indicated by RT-PCR (Figure 5(e)), Western blot (Figure 5(f)), and IHC (Figure 5(g)), but AAV-LacZ had no effects.

### 3.5. Oxidative Stress Promotes Inflammation and Inhibits Mitophagy in Myocytes

**Figure 6:**
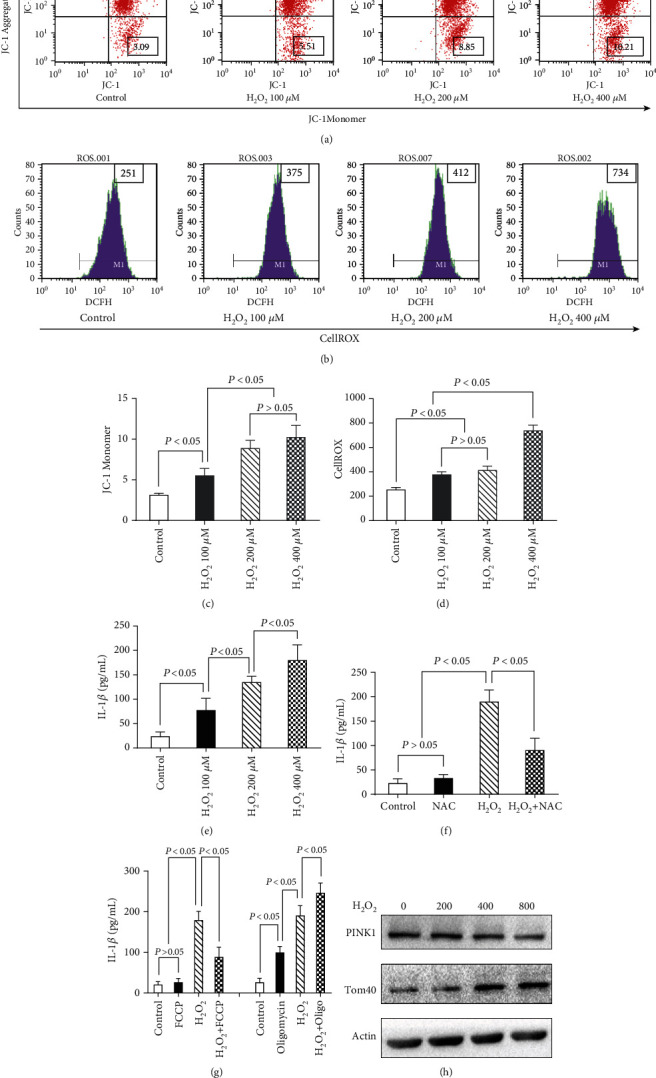
H_2_O_2_ increases MMP and ROS and activates inflammatory responses in myocytes. Detection of MMP (a, c) and cellular oxidative stress (b, d) in H_2_O_2_ (0-400 *μ*M, 4 h) treated myocytes. Secretion of IL-1*β* in myocytes treated by H_2_O_2_ (0-400 *μ*M, 4 h) (e), secretion of IL-1*β* in myocytes treated by H_2_O_2_ (400 *μ*M, 4 h) together with NAC (10 mM) (f), FCCP (10 *μ*M) or oligomycin (10 *μ*M) (g). Western blot revealed the expression of PINK1 and Tom40 in myocytes treated by H_2_O_2_ (0-400 *μ*M) (h).

The effect of oxidative stress on myocytes was then investigated. Over one hour, H_2_O_2_ (0-400 *μ*M) administration lowered mitochondrial membrane potential (MMP) (Figures 6(a) and 6(c)) and increased ROS accumulation (Figures 6(b) and 6(d)) and inflammatory responses (Figure 6(e)) in a dose-dependent manner. Furthermore, the antioxidant N-acetylcysteine (NAC) (10 mM) effectively suppressed H_2_O_2_- (400 *μ*M-) induced IL-1*β* secretion in myocytes, suggesting that ROS plays a key role in myocyte inflammation (Figure 6(f)).

Recent research has found that mitophagy reduces inflammation by blocking the NLRP3 inflammasome. As a result, we investigated whether ROS could trigger inflammatory responses by blocking mitophagy. The use of oligomycin (10 *μ*M), a mitophagy inhibitor, enhanced H_2_O_2_-induced IL-1*β* production; moreover, the use of FCCP (10 *μ*M), a medication that dissipates MMP and induces mitophagy by activating PINK1, protected myocytes against H_2_O_2_-induced inflammatory responses (Figure 6(g)). Because ROS-induced inflammatory responses in myocyte were modulated by mitophagic inhibitor and inducer, we further examined whether ROS regulated mitophagy in myocytes. As the initiator of mitophagy, PINK1 phosphorylates ubiquitin to activate Parkin, which builds ubiquitin chains on mitochondrial outer membrane proteins. Incubation with H_2_O_2_ (0-400 *μ*M, 4 h) decreased PINK1 expression in a dose-dependent manner, according to our findings. Generally, the amount of Tom40 protein rises when mitophagy is inhibited. Our results showed that Tom40 accumulation was enhanced by H_2_O_2_ in a dose-dependent manner (Figure 6(h)).

### 3.6. T*β*4 Attenuated H_2_O_2_-Induced Mitophagy Inhibition and Inflammasome Activation and Promoted Proliferation in Myocytes

**Figure 7:**
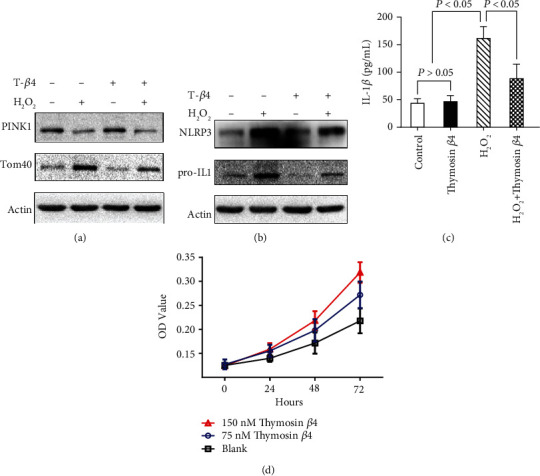
T*β*4 reduces H_2_O_2_-induced inhibition of mitophagy and inflammasome activation and promotes proliferation in myocyte. Western blot revealed the expression of PINK1 and Tom40 (a) and NLRP3 and IL-1*β* (b) in myocytes treated by T*β*4 (150 nM, 4 h) and/or H_2_O_2_ (400 *μ*M). Secretion of IL-1*β* in myocytes treated by T*β*4 (150 nM, 4 h) and/or H_2_O_2_ (400 *μ*M) (c). Development of myocytes treated by T*β*4 (75-150 nM, 12 h) (d).

We first looked at how T*β*4 influences mitophagy and inflammatory responses in myocytes; T*β*4 (150 nM, 4 h) reversed the effects of H_2_O_2_ (400 *μ*M) on the expression of PINK1 and the accumulation of Tom40 (Figure 7(a)). In myocytes, T*β*4 (150 nM, 4 h) inhibited NLRP3 inflammasome activation and IL-1*β* secretion induced by H_2_O_2_ (400 *μ*M) (Figures 7(b) and 7(c)). T*β*4 (75-150 nM, 12 h) promoted the development of myocytes in a dose-dependent manner based on a CCK-8 assay (Figure 7(d)).

### 3.7. T*β*4 Suppresses the Proliferation and Attenuates the TGF-*β*1-Induced Activation of Myofibroblasts

**Figure 8:**
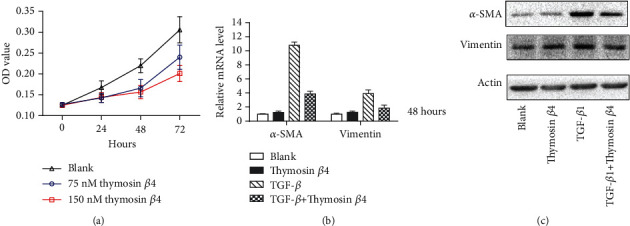
T*β*4 inhibits myofibroblast growth and TGF-*β*1-induced activation. Development of myofibroblast cells treated by T*β*4 (75-150 nM, 12 h) (a). Expression of *α*-SMA and vimentin in myofibroblast cells treated by T*β*4 (150 nM, 12 h) and/or TGF-*β*1 (5 ng/mL) at mRNA (b) and protein (c) levels.

T*β*4 (75-150 nM, 12 h) significantly inhibited myofibroblast growth based on a CCK-8 assay in dose-dependent manner (Figure 8(a)). According to RT-PCR, T*β*4 (150 nM, 12 h) did not influence in basal expression of *α*-SMA and vimentin in myofibroblast, but it dramatically decreased the elevation of *α*-SMA and vimentin induced by TGF-*β*1 (5 ng/mL) (Figures 8(b) and 8(c)).

## 4. Discussion

In the present study, we first provided substantial evidence for the increased expression of T*β*4 in murine models of ligation-induced AMI and cardiac fibrosis. The role of T*β*4 in alleviating hepatic, renal, and cardiac injury and fibrosis has been confirmed in recent studies [16, 26]. The increased production of local T*β*4 in mice is an adaptive response to heart injury, but this increased expression of endogenous T*β*4 might not be sufficient to alleviate heart injury and fibrosis. In the present study, we observed the effects of adeno-associated virus-mediated T*β*4 ectopic expression on ligation-induced AMI and subsequent cardiac fibrosis. Our findings indicated a protective role of T*β*4 against oxidant damage and inflammasome activity, thereby alleviating myocardial infarction and cardiac fibrosis.

Previous studies have revealed the role of oxidative stress in the pathogenesis of cardiac inflammatory responses; the generation of mitochondrial ROS is crucial for NLRP3 inflammasome activation, leading to the release of IL-1*β* [27]. Under inflammatory conditions, infiltrated and activated inflammatory cells, such as neutrophils, monocytes/macrophages, and eosinophils, can generate ROS via multiple enzymes and reaction pathways, including nicotinamide adenine dinucleotide phosphate oxidases, eosinophil peroxidase, and especially MPO. MPO catalyzes the formation of potent cytotoxic oxidants. The relationship between myocytes, oxidative stress, and inflammation then creates a vicious cycle. Here, our in vitro data demonstrated that ROS promoted inflammation in myocytes, which is consistent with previous findings that myocyte injury leads to the secretion of IL-1*β* [28]. We also found that H_2_O_2_ treatment induced ROS generation in myocytes, thereby leading to the activation of the NLRP3 inflammasome, but this effect was alleviated by NAC, an antioxidant.

Although debates exist, inflammation is believed to contribute to the pathological progress of AMI and cardiac fibrosis, especially during the initial period. Infiltration of leukocytes into the heart leads to myocyte dysfunction and tissue damage, which trigger fibrogenic progression. Moreover, infiltrated leukocytes and damaged tissue cells can release proinflammatory cytokines, such as IL-1*β*, TNF-*α*, and IL-6, which also exert fibrogenic effects. Similarly, our present study demonstrated that T*β*4 alleviated ligation-induced heart inflammation as well as the production of profibrotic cytokines, suggesting that the anti-inflammatory potency of T*β*4 contributed to its antifibrotic effect.

Excessive ROS production is a hallmark of many diseases; dysfunctional mitochondria have been implicated in these disorders, acting as both a source and a target of ROS [29]. Mitophagy is a kind of selective autophagy in which damaged or undesired mitochondria are degraded. In this study, myocyte mitophagy was discovered to be impaired, and NAC was found to alleviate this condition. Furthermore, we discovered that FCCP, a mitophagy inducer, inhibited H_2_O_2_-induced IL-1*β* production in myocytes, whereas oligomycin, a mitophagy inhibitor, enhanced production. Defective mitophagy leads to the buildup of damaged ROS-generating mitochondria and the activation of the NLRP3 inflammasome. Our data revealed for the first time that ROS promotes inflammation via mitophagy inhibition in myocytes.

T*β*4 has been shown to have antioxidant and anti-inflammatory properties [14, 22]. AAV-T*β*4 induced exogenous expression of T*β*4, which effectively reduced infarction-induced increases in mouse cardiac MPO activity, MDA levels, and proinflammatory cytokines in vivo, according to our findings. Inflammation is thought to have a role in the initial etiology of myocardial infarction and cardiac fibrosis; myocyte dysfunction and subsequent inflammation initiate the fibrogenic process, which results in matrix deposition and heart remodeling [30]. Our data demonstrated that exogenous T*β*4 reduced infarction-induced myocardial damage and cardiac fibrosis in mice and decreased the fibrogenic process in myofibroblasts. In addition to protecting the heart from oxidative injury, our study demonstrated that T*β*4 promoted myocyte growth but attenuated myofibroblast growth. T*β*4 is a key factor in cardiac development, growth, disease, epicardial integrity, and blood vessel formation and has cardio-protective properties. The proliferation-promoting effect of T*β*4 might facilitate the repair of damaged myocytes in a manner that avoids aberrant repair leading to fibrosis. In contrast to promoting the proliferation of myocytes, T*β*4 suppressed the growth of myofibroblasts, which should inhibit the accumulation of ECM and further alleviate fibrogenesis.

In conclusion, our study demonstrated that elevated expression of T*β*4 during heart injury and inflammation serves as a counteracting mechanism to protect against subsequent fibrogenesis. The protective effects of T*β*4 may include the reduction of oxidative stress, the upregulation of mitophagy, and the reduction of inflammation. Supplementation of exogenous T*β*4 or enhancing its endogenous expression should be therapeutically beneficial not only to manage infarction-induced myocardial infarction but also to relieve cardiac fibrosis.

## Figures and Tables

**Table 1:** AAV-T*β*4 improves cardiac function of AMI mice.

	LVIDd (mm)	IVS (mm)	LVIDs (mm)	LVEF (%)
NS	7.93 ± 0.28	1.36 ± 0.07	4.50 ± 0.28^∗^	79.3 ± 2.6^∗^
AMI	8.20 ± 0.32	1.36 ± 0.10	5.50 ± 0.48	66.8 ± 5.4
AMI+AAV-LacZ	8.27 ± 0.21	1.31 ± 0.09	5.30 ± 0.45	70.0 ± 5.8
AMI+AAV-T*β*4	8.00 ± 0.21	1.35 ± 0.10	4.90 ± 0.24^∗^	74.3 ± 3.2^∗^

^∗^ *P*<0.05 vs the AMI or AMI+AAV-LacZ group.

**Table 2:** AAV-T*β*4 alleviates increases in MDA content and MPO activity in heart tissues.

	MDA	(nmol/mg protein)	MPO	(U/mg protein)
	Day 7	Day 42	Day 7	Day 42
NS	0.71 ± 0.66 (*n* = 7)^∗^	0.82 ± 0.36 (*n* = 7)^∗^	0.48 ± 0.42 (*n* = 7)^∗^	0.66 ± 0.25 (*n* = 7)^∗^
AMI	1.45 ± 0.43 (*n* = 7)	1.88 ± 0.85 (*n* = 4)	0.92 ± 0.23 (*n* = 7)	1.27 ± 0.89 (*n* = 4)
AMI+AAV-LacZ	1.48 ± 0.33 (*n* = 7)	1.97 ± 0.86 (*n* = 5)	0.89 ± 0.35 (*n* = 7)	1.12 ± 0.93 (*n* = 5)
AMI+AAV-T*β*4	0.91 ± 0.27 (*n* = 7)^∗^	1.18 ± 0.41 (*n* = 6)^∗^	0.55 ± 0.12 (*n* = 7)^∗^	0.78 ± 0.23 (*n* = 6)^∗^

^∗^ *P*<0.05 vs the AMI or AMI+AAV-LacZ group.

**(a):** 

Day 7	*n*	IL-1*β* (pg/mL)	IL-6 (pg/mL)	TNF-*α* (pg/mL)
NS	7	32.29 ± 2.92^∗^	34.29 ± 3.29^∗^	3.87 ± 0.38^∗^
AMI	7	55.86 ± 5.16	59.29 ± 5.17	6.36 ± 0.50
AMI+AAV-LacZ	7	51.57 ± 5.69	58.71 ± 5.58	6.41 ± 0.56
AMI+AAV-T*β*4	7	38.71 ± 4.02^∗^	40.71 ± 3.90^∗^	3.39 ± 0.41^∗^

**(b):** 

Day 42	*n*	IL-1*β* (pg/mL)	IL-6 (pg/mL)	TNF-*α* (pg/mL)
NS	7	64.32 ± 3.24^∗^	75.56 ± 2.45^∗^	7.02 ± 2.21^∗^
AMI	4	89.41 ± 3.98	95.43 ± 4.48	18.76 ± 1.96
AMI+AAV-LacZ	5	90.13 ± 4.65	89.33 ± 3.32	16.53 ± 3.94
AMI+AAV-T*β*4	6	66.41 ± 5.54^∗^	78.89 ± 3.76^∗^	9.09 ± 1.97^∗^

^∗^ *P*<0.01 vs the AMI or AMI+AAV-LacZ group.

## Data Availability

On reasonable request, the corresponding author will provide the datasets used and/or analyzed during the current work.

## Abbreviations

ECM: Extracellular matrix
AMI: Acute myocardial infarction
T*β*4: Thymosin 4
ROS: Reactive oxygen species
SOD: Superoxide dismutase
AAV: Adeno-associated virus
NS: Normal sham
MDA: Malondialdehyde
MPO: Myeloperoxidase
*α*-SMA: *α*-Smooth muscle actin
MMP: Mitochondrial membrane potential.
